# Large language models in health care: Development, applications, and challenges

**DOI:** 10.1002/hcs2.61

**Published:** 2023-07-24

**Authors:** Rui Yang, Ting Fang Tan, Wei Lu, Arun James Thirunavukarasu, Daniel Shu Wei Ting, Nan Liu

**Affiliations:** ^1^ Department of Biomedical Informatics, Yong Loo Lin School of Medicine National University of Singapore Singapore Singapore; ^2^ Singapore National Eye Center, Singapore Eye Research Institute Singapore Health Service Singapore Singapore; ^3^ StatNLP Research Group Singapore University of Technology and Design Singapore; ^4^ University of Cambridge School of Clinical Medicine Cambridge UK; ^5^ Duke‐NUS Medical School Centre for Quantitative Medicine Singapore Singapore; ^6^ Duke‐NUS Medical School Programme in Health Services and Systems Research Singapore Singapore

**Keywords:** Large language model, AI, Health care

## Abstract

Recently, the emergence of ChatGPT, an artificial intelligence chatbot developed by OpenAI, has attracted significant attention due to its exceptional language comprehension and content generation capabilities, highlighting the immense potential of large language models (LLMs). LLMs have become a burgeoning hotspot across many fields, including health care. Within health care, LLMs may be classified into LLMs for the biomedical domain and LLMs for the clinical domain based on the corpora used for pre‐training. In the last 3 years, these domain‐specific LLMs have demonstrated exceptional performance on multiple natural language processing tasks, surpassing the performance of general LLMs as well. This not only emphasizes the significance of developing dedicated LLMs for the specific domains, but also raises expectations for their applications in health care. We believe that LLMs may be used widely in preconsultation, diagnosis, and management, with appropriate development and supervision. Additionally, LLMs hold tremendous promise in assisting with medical education, medical writing and other related applications. Likewise, health care systems must recognize and address the challenges posed by LLMs.

## Graphical abstract

The advancement of large language models (LLMs) promises to bring significant changes in health care, with extensive applications spanning pre‐consultation, diagnostic, and management phases. Moreover, LLMs hold potential utility in medical education and medical writing. Concurrently, it is imperative to recognize the limitations of LLMs, strengthen review measures and enhance supervision system, thereby facilitating more effective collaboration between related personnel and LLMs.

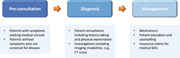

**Abbreviations**
AI: artificial Intelligence
BERT: Bidirectional Encoder Representations from Transformers
BioBERT: Bidirectional Encoder Representations from Transformers for Biomedical Text Mining
CAD: computer‐aided diagnosis
EHR: electronic health records
GPT: Generative Pretrained Transformer
LLaMA: large language model meta AI
LLMs: large language models
NLP: nature language processing
PaLM: Pathways Language Model
PMC: PubMed central
USMLE: United States Medical Licensing Examinations

## 1 INTRODUCTION

The field of natural language processing (NLP) has seen significant advances with the development of large language models (LLMs) trained by deep neural networks using massive text datasets, generally with billions of parameters. In 2017, Google first demonstrated “Transformer” architecture for the task of machine translation, which later attained state‐of‐the‐art performance in many NLP tasks [1]. Since then, many LLMs with “Transformer” architecture have been developed, such as Bidirectional Encoder Representations from Transformers (BERT) [2], Generative Pretrained Transformer‐3 (GPT‐3) [3], Pathways Language Model (PaLM) [4], LLM Meta AI (LLaMA) [5], and GPT‐4 [6].

In general, LLMs may be subcategorized based on their pre‐training architecture as either encoder‐only, decoder‐only, or encoder‐decoder; the pre‐training tasks they undertake or the datasets utilized during their training phase [7]. As the size and computational resources used to train LLMs have increased, “zero‐shot” or “few‐shot” performance has experienced significant enhancements. When faced with new tasks, models are able to learn and accomplish these novel tasks without prior specialized training, simply by being shown zero or a few examples of these tasks [8]. The rapid development and outstanding performance of LLMs in NLP tasks may result in profound changes to health care work, although barriers to implementation must be overcome [9].

Already, NLP technology has been applied in health care to support preconsultation, diagnosis and management [10, 11, 12]. Development and proliferation of LLMs will improve NLP aptitude, and may therefore allow artificial intelligence (AI) to have an even greater impact on clinical care: changing how consultation, diagnosis, and management are conducted, and enhancing accessibility and autonomy by improving the provision of patient education through interactive dialogue with biomedical or clinical language models [13, 14, 15, 16, 17, 18]. LLMs also have the potential to assist medical education for clinicians and administrative tasks such as writing letters, clinic notes, and discharge summaries [19, 20, 21].

In this review, we provide an overview of the development of LLMs designed for biomedical or clinical use. We subsequently explore the potential and trialed applications of LLMs in clinical contexts. Finally, we outline the challenges and limitations which must be addressed to ensure that LLM technology realizes its clinical potential.

## 2 DEVELOPMENT OF LLMs IN HEALTH CARE

Although LLMs have shown impressive performance across a range of NLP tasks, their efficacy in specialized tasks is limited [22]. A lack of domain‐specific knowledge in general LLMs hinders their ability to interpret technical terms and produce accurate, reasoned answers. Moreover, there are significant differences between general corpora and professional corpora, which further hinder the ability of LLMs to perform well in biomedical or clinical tasks [23]. To improve domain‐specific performance by addressing these weaknesses, specialized LLMs have been developed.

BERT for Biomedical Text Mining (BioBERT) was built using a large biomedical corpus of PubMed abstracts and PubMed Central full‐text articles for fine‐tuning [13]. SCIBERT was trained from scratch on the Semantic Scholar corpora (18% computer science papers and 82% biomedical papers), rather than fine‐tuning the generalist BERT model [15]. PubMedBERT was developed through a similar schema to SCIBERT, but using corpora sourced entirely from PubMed [16]. Compared to BERT, BioBERT, SCIBERT and PubMedBERT demonstrated superior performance in biomedical NLP tasks. However, clinical use cases of these biomedical language models are limited.

ClinicalBERT was created based upon BERT and BioBERT architectures [14] and trained on the MIMIC‐III data set [24]. MIMIC‐III comprises demographics, vital signs, laboratory tests, procedures, medications, clinical notes, investigation reports, and mortality data corresponding to over 40,000 critical care patients—a rich source of domain‐specific information [24]. ClinicalBERT attained superior performance to BERT and BioBERT across a range of medical NLP tasks, demonstrating the promise of using clinical corpora to fine‐tune LLMs to optimize domain‐specific performance [14]. In addition, GatorTron, the largest clinical language model available, was trained from scratch using over 90 billion words of text from the deidentified clinical notes of University of Florida Health, PubMed articles and Wikipedia [25]. This model increases the parameter count of LLMs within the clinical domain from 110 million (ClinicalBERT) to 8.9 billion. It has achieved competitive performance across multiple downstream clinical tasks, demonstrating the advantage of using large “Transformer” models.

Moreover, the emerging conversational abilities of LLMs have fostered innovation in health care. The performance of PaLM in medical questions was optimized through instruction prompt tuning to develop Med‐PaLM [26] (ChatGPT‐like ChatBot for Health care). Subsequent development at Google has resulted in the production of Med‐PaLM 2 [27], which reportedly achieves state‐of‐the‐art performance in United States Medical Licensing Examinations (USMLE) questions, exceeding the performance of ChatGPT [20]. ChatDoctor (an open‐source Chatbot for health care) was based on LLaMA and used 100,000 patient‐physician conversations to fine‐tune [28]; this model showed significant improvements in understanding patients' needs and providing accurate advice. Similarly, Baize‐health care [29] is another open‐source Chatbot for health care based on LLaMA, which has been fine‐tuned using MedQuAD data set [30] (including 46,867 medical dialogues) and performed well in multi‐turn conversations. These models will facilitate the further development of conversation models in health care.

While the performance of general LLMs is impressive, and advancing rapidly, domain‐specific models may remain optimal for specialized tasks. Rather than training domain‐specific models from the ground up, further research may seek to fine‐tune or prompt‐tune these general LLMs to optimize performance in particular tasks. Using larger open‐source base models and newer interactive LLMs could further improve the capabilities of decentralized researchers around the world, who could then fine‐tune LLMs to optimize performance for clinical work. Through bespoke fine‐tuning, domain‐specific LLMs may be produced to serve narrowly defined, well‐specified tasks—minimizing error and maximizing clinical utility. Whether developed from scratch or fine‐tuned using existing models, LLM applications will become more sophisticated and begin to impact patients and practitioners at scale.

## 3 APPLICATIONS OF LLMs IN HEALTH CARE

**Figure 1:**
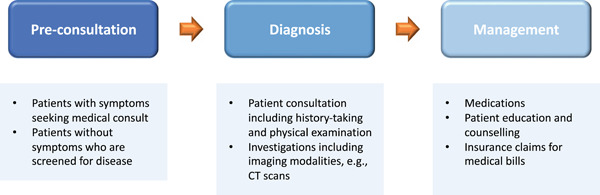
Potential touch points along a patient's care journey for the application of large language models.

A typical patient journey in health care (as outlined in Figure 1) includes: (1) Preconsultation: where patients register for medical consult or undergo health screening; (2) Diagnosis: which includes patient consultation and examination as well as adjunctive investigations; and (3) Management: which includes medications, patient counseling and education, and reimbursements for medical bills. LLMs show promise to enhance the patient experience at each of these touch points in the patient journey.

### 3.1 Preconsultation

To cope with the exponential increase in patient load, LLMs can facilitate triaging patients and directing efficient use of resources. By combining with cloud services, a cloud‐based intelligent self‐diagnosis and department recommendation service built on LLM was able to predict the possible disease categories based on patients' history of presenting complaints, and then recommended the specific medical subspecialty for patients to book a doctor's appointment [17]. This provided a recommendation framework for patients to seek appropriate medical review, and minimized wastage of medical resources. Furthermore, LLMs can also be integrated with remote diagnosis systems or telehealth services to enhance access to care for patients facing geographical barriers or mobility limitations. LLMs can serve as real‐time self‐assessment tools via SMS or other platforms on mobile devices for individuals in remote settings like sub‐Saharan Africa [12]. This tool provided timely access to health care by assessing symptoms of tropical diseases, suggesting a likely diagnosis, and providing medical advice. In addition to assessing patients' symptoms, LLMs can improve predictions in the context of patients' medical background by harnessing additional information like comorbidities, risks factors and medication lists.

### 3.2 Diagnosis

Patient consultation, especially the first encounter, is important not only in the diagnostic process but essential in laying the foundation of the patient‐physician relationship. However, the reality of clinical practice is that appointments are often overbooked with minimal consultation time for each patient. Furthermore, each consultation consists of reviewing electronic health records (EHR), history‐taking, physical examination, and patient counseling. Therefore LLMs can be leveraged to generate concise summaries of each patient's medical background including comorbidities, previous consultations or admissions, medication lists, past treatment progress and response [11, 18, 25]. This synthesizes and brings to the physician's attention relevant patient information that may be essential in disease diagnosis. This also facilitates a more efficient and comprehensive consultation, allowing the physician to dedicate more time for patient interaction and lead to greater patient satisfaction.

In the diagnostic process, adjunctive investigations are often required to support clinical suspicion and confirm the diagnosis. LLMs can serve as clinical decision support systems to guide physicians to select the most appropriate radiological investigations in specific clinical scenarios [18]. This can minimize unnecessary burden on existing resource capacity, especially in public hospitals or low‐resource settings, where imaging modalities and technical support are limited. Moreover, patients who may not require contrast computed tomography scans, for example, can avoid having unnecessary radiation or contrast exposure.

There have been increasing applications of deep learning models utilized to build computer‐aided diagnosis (CAD) systems to automate efficient and highly accurate interpretation of these medical imaging modalities for disease detection and classification. However, most clinicians remain skeptical and hesitant to adopt these into real‐world clinical practice, attributed to the opaque model decision‐making processes. By incorporating LLMs into these CAD systems, clinicians can ask open‐ended questions about specific input images or patients to understand the rationale of the CAD's decision output [18]. This human‐computer interaction could potentially enhance model interpretability and encourage uptake into existing diagnostic workflows. Furthermore, clinicians may even uncover new insights or imaging biomarkers of disease in the process.

### 3.3 Management

Optimal management requires a multidisciplinary approach with an increasing emphasis on people‐centered care to empower patients to participate and take ownership of their own health [31]. Medication compliance is a significant challenge affecting treatment effectiveness, often attributed to forgetfulness and poor patient insight. Engaging patients through patient education initiatives may boost compliance and encourage patients to be responsible for their own health. LLMs may enable tailored patient education to improve understandability and engagement. Recently, Macy, an AI pharmacist comprising a photorealistic animated avatar incorporating ChatGPT, was capable of delivering medication counseling via video. This was structured in plain‐language and included essential information like medication dosage and frequency, precautions and red flag symptoms to watch out for. While solely experimental, it was developed in under 30‐min and at no significant cost, demonstrating the potential of LLMs to revolutionize patient education and beyond [32].

LLMs can also generate educational content at an appropriate level for patients, such as postprocedure counseling, medication counseling, and lifestyle modifications [33]. This allows complex medical terminology to be communicated effectively and appropriately to patients in simple terms to facilitate understanding. For example, LLMs can be used for autocomplete text simplification tasks, to translate jargon‐heavy medical reports or explanations into simplified sentences by prompting simple words to follow what has been typed by the physician [34]. This expedites the process of text simplification while preserving control of the information translated, allowing the physician to ensure quality and accuracy of the information communicated. LLMs can also efficiently automate multilingual translations to cater to a wider diversity of patients. Another example would be AnsibleHealth, a virtual clinic for chronic lung diseases, which explored the use of ChatGPT to simplify radiology reports and jargon‐heavy medical records to facilitate patient comprehension [19].

Managing patients with mental health conditions is challenging, requiring multimodal and multidisciplinary approaches. LLMs can potentially be effective in addressing the clinical need for access to psychiatric care services and treatment, supplementing the shortage of health care professionals and enhancing patient compliance [35]. For example, Woebot is a fully automated text‐based conversational assistant that delivers cognitive behavior therapy services for adolescents with depression. Woebot significantly reduced symptoms of depression, compared to the control group who was given information‐only e‐book materials [35, 36]. SERMO is another conversational tool that guides patients with mental health conditions in regulating their emotions to better handle negative thoughts [37]. It automatically detects the type of emotion based on user text inputs, and recommends mindfulness activities or exercises tailored to the specific emotions.

Furthermore, LLMs have the potential to streamline administrative processes to increase efficiency while reducing the administrative burden on physicians and enhancing patient experience. This can encompass drafting discharge summaries and operation reports, extracting succinct clinical information from EHR to complete medical reports and translating them into billable codes for reimbursement claims, as well as automating responses to general patient queries (e.g., requests for medication top‐up, appointment booking and rescheduling) [38, 39, 40].

### 3.4 Medical education and medical writing

In addition to health care applications from the patient perspective, LLMs hold immense potential in reshaping medical education and research. Existing LLMs have been able to pass undergraduate and postgraduate medical examinations [19, 41, 42]. Moreover, answers generated by ChatGPT to USMLE were accompanied by justifications that have a high level of concordance and offered new insights [17, 31]. The logical flow of explanations and deductive reasoning with additional supplementary information provided allows students to easily follow and comprehend. For example, this can be targeted at an undergraduate medical student who may have answered the question incorrectly, to uncover new perspectives or remedial knowledge from the ChatGPT‐generated explanations. ChatGPT can also suggest innovative and unique mnemonics to aid memorizing. The interactive interface of LLMs can complement existing student‐directed learning, where Socratic style of teaching has been surveyed as preferable by students over didactic lectures [20, 43, 44].

LLMs can also add value to medical research. LLMs can improve the efficiency of research article writing by automating tasks such as literature review, generating text and guiding manuscript writing style and formatting [44]. Biswas recently published a perspective piece that was written by ChatGPT, though still requiring editing by a human author [45, 46]. LLMs can also match patients to potential clinical trial opportunities relevant to patients' conditions and within inclusion and exclusion criteria. This can facilitate research patient recruitment, while enabling access to potentially breakthrough treatments that may not be otherwise available or affordable for patients [45, 46].

## 4 CHALLENGES OF LLMs IN HEALTH CARE

### 4.1 Data privacy

One of the challenges in validation and implementation of LLMs with real‐world clinical patient data would be the risk of leaking confidential and sensitive patient information. For example, adversarial attacks on a LLM GPT‐2 were successful in extracting the model's training data [47, 48]. By querying GPT‐2 structured questions, training data including personal identifiable information and internet relay chat conversations were extracted verbatim. Moreover, despite anonymizing sensitive patient health information, some algorithms demonstrated the capability to reidentify these patients [49, 50, 51]. To mitigate these challenges, possible strategies include pseudonymization or filtering patient identifiers, differential privacy, and auditing of LLMs using data extraction attacks [47, 48, 52].

### 4.2 Questionable credibility and accuracy of information

Some have criticized LLMs for the questionable credibility and accuracy of information generated. Open domain nonspecific LLMs may be at risk of perpetuating inaccurate information from open internet sources, or generalize poorly across different contextual settings [47, 48, 52, 53]. The term “hallucination effect” has been used to describe trivial guessing behaviors observed in LLMs [54]. For example, an experiment using GPT‐3.5 to answer sample medical questions from USMLE, found that the model often predicted options A and D. In the ChatGPT‐generated perspective article, three fabricated citations were identified during editing by the human author [45]. This may potentially be hazardous to users who are unable to discern seemingly credible but inaccurate or misleading answers. Despite its potential as an educational tool and source of information for patients, medical students, and the research community, human oversight and additional quality measures are essential in ensuring accuracy and quality control of the generated content.

### 4.3 Data bias

LLMs are commonly trained on vast and diverse data which is often biased. Consequently, the content generated by LLMs may perpetuate and even amplify bias, such as ethnicity, gender, and socioeconomic background [55]. These biases are especially problematic in health care, where differential treatment may lead to exacerbation of disparities in mortality and morbidity. For example, a study focusing on skin cancer may predominantly involve participants with fair‐skinned individuals, resulting in an LLM that is less adept at identifying skin cancer in those with darker‐skinned individuals. This could lead to misdiagnosis and delayed or inappropriate treatments, further widening health disparities. The absence of minority groups in training data may make LLMs exacerbate these biases, leading to inaccurate results. Moving towards fair artificial intelligence and combating bias will be a significant challenge for LLMs [56].

### 4.4 Interpretability of LLMs

The lack of interpretability of the decision‐making process of LLMs remains a barrier to adoption into clinical practice [57]. LLM‐generated responses are largely not accompanied by justifications or supporting information sources. This is further exacerbated by the tendency of LLMs to fabricate facts in a seemingly confident manner or rely on trivial guessing, as elaborated above. In the context of safety‐critical tasks in health care, this may limit trust and acceptance by physicians and patients, where the consequences of delivering inaccurate medical advice may be detrimental. Proposed methods to improve interpretability include a selection inference multi‐step reasoning framework by Creswell et al. to generate a series of casual reasoning steps toward the final generated response [58]. Another method proposed leveraging ChatGPT using chain‐of‐thought prompting (i.e. step‐by‐step instructions) [59] for knowledge graph extraction, where extracted entities and relationships from the raw input text are presented in a structured format, which was then used to train an interpretable linear model for text classification [60]. Uncertainty‐aware LLM applications may be another useful feature, where differential weights of input data or reporting confidence scores of generated responses can enhance the trust in proposed LLM applications [61].

### 4.5 Roles of LLMs

Another challenge LLMs may face lies in defining its role and identity in scientific research and clinical practice [62]. Questions that may arise include: Can AI be a researcher or a physician? Can the AI be responsible for the content it generates? How to distinguish the text generated by AI versus humans? What to do when physicians have different views than AI? It is worth noting that LLMs may fabricate false content, so it is necessary to avoid overusing [55]. From preconsultation to diagnosis to treatment, or in medical education, medical research, LLMs can serve in complementary roles rather than substitutes for physicians. Although LLMs can undergo self‐improvement, physician oversight is still required to ensure the generated content is accurate and clinically relevant.

### 4.6 Deployment of LLMs

LLaMA's open source facilitates the deployment of LLMs on resource‐constrained devices, such as laptops, phones, and Raspberry Pi systems [5]. Alpaca's fine‐tuning based on LLaMA, enables the rapid (within hours) and cost‐effective (costing under US$600) development of models that exhibit performance comparable to that of GPT‐3.5 [63]. This makes it possible to train personalized language models with high performance at a reduced cost, but it is important to recognize that these models also inherit various biases. When applied to general purposes, they may generate harmful or sensitive content, potentially compromising user security. Furthermore, the ease of deployment may increase the likelihood of LLMs being misused or even maliciously trained to disseminate deeply falsified information and detrimental content. Such outcomes could undermine public trust in AI and have deleterious effects on the whole society. To ensure that LLMs are harnessed for their intended purposes and to reduce the risks associated with their misuse, it is crucial to develop and implement various safeguards. These may include technical solutions for filtering out sensitive and harmful content, the establishment of stringent terms of use and deployment specifications. By adopting such measures, the potential dangers of deploying LLMs on small personal devices can be effectively controlled.

### 4.7 Clinical domain‐specific LLMs

There is no doubt that LLMs are having a significant impact in the health care field. Regardless of whether it is preconsultation, diagnosis, management, medical education, or medical writing, all these areas will undergo transformative changes due to the development of LLMs. In this regard, it is essential to recognize that when LLMs are deployed in real clinical settings, different medical specialties will encounter a variety of unique challenges [64]. For example, the type and quality of data may differ significantly between domains. Additionally, the diverse application scenarios and tasks of LLMs will lead to inconsistencies in the standards expected by clinical professionals. In light of this, when deploying LLMs in clinical environments, we should recognize the variations across clinical specialties and make appropriate adjustments according to the specific application scenarios.

## 5 CONCLUSION

LLMs are poised to bring about significant transformation in health care and will be ubiquitous in this field. To make LLMs more serviceable for health care, training from scratch with medical databases or fine‐tuning with generic LLMs would be effective approaches. Besides, LLMs can further perform multimodal feature fusion with diverse data sources, including image data and tabular data, resulting in better performance, even beyond human level. While the use of LLMs presents numerous benefits, we should recognize that LLMs cannot take full responsibility for generated content. It is essential to ensure that AI‐generated content is properly reviewed to avoid any potential harm. As the threshold for the deployment of LLMs diminishes, improving deployment specifications also deserves attention. Simultaneously, efforts should be made to promote the integration of LLMs in clinical practice, improve the interpretability of LLMs in clinical settings and enhance human‐machine collaboration to better support clinical decision‐making. By leveraging LLMs as a complementary tool, physicians can maximize the benefits of AI while mitigating potential risks and achieve better clinical outcomes for patients. Ultimately, the successful integration of LLMs in health care will require the collaborative efforts of physicians, data scientists, administrators, patients, and regulatory bodies.

## AUTHOR CONTRIBUTIONS

**Rui Yang**: Conceptualization (equal); writing—original draft (equal); writing—review and editing (equal). **Ting Fang Tan**: Conceptualization (equal); writing—original draft (equal); writing—review and editing (equal). **Wei Lu**: Conceptualization (equal); writing—review and editing (equal). **Arun James Thirunavukarasu**: Conceptualization (equal); writing—review and editing (equal). **Daniel Shu Wei Ting**: Conceptualization (equal); supervision (lead); writing—review and editing (equal). **Nan Liu**: Conceptualization (lead); project administration (lead); supervision (lead); writing—original draft (supporting); writing—review and editing (equal).

## CONFLICT OF INTEREST STATEMENT

The authors declare no conflicts of interest.

## ETHICS STATEMENT

Not applicable.

## INFORMED CONSENT

Not applicable.

## Data Availability

Not applicable.
